# Identification and functional analysis of a novel missense mutation in GJA8, p.Ala69Thr

**DOI:** 10.1186/s12886-020-01725-1

**Published:** 2020-11-20

**Authors:** Dandan Li, Chenjia Xu, Dandan Huang, Ruru Guo, Jian Ji, Wei Liu

**Affiliations:** 1 Department of Ophthalmology, Tianjin TEDA Hospital, 300457 Tianjin, China; 2 Tianjin Key Laboratory of Retinal Functions and Diseases, Tianjin International Joint Research and Development Centre of Ophthalmology and Vision Science, Eye Institute and School of Optometry, Tianjin Medical University Eye Hospital, 251 Fukang Road, Nankai District, Tianjin, 300384 China; 3 Department of Ophthalmology, Taihe Hospital, Hubei University of Medicine, Shiyan, Hubei China

**Keywords:** *GJA8*, Congenital cataract, Hemichannel

## Abstract

**Background:**

To explore the molecular genetic cause of a four-generation autosomal dominant congenital cataract family in China.

**Methods:**

Targeted region sequencing was performed to screen for the potential mutation, and Sanger sequencing was used to confirm the mutation. The homology model was constructed to identify the protein structural change, PolyPhen-2 and Provean were used to predict the mutation impact. Functional and cellular analysis of the wild and mutant *GJA8* were performed in DF-1 cells by western blotting, dye uptake assay, immunofluorescence, Annexin V-FITC staining.

**Results:**

A novel heterozygous mutation (c.205G > A; p.Ala69Thr) was identified within *GJA8*, which cosegregated with congenital cataract phenotype in this family. Bioinformatics analysis showed the mutation was located in a highly conserved region, and the mutation was predicted to be pathogenic. Function analysis indicated that the mutation inhibited *GJA8* hemichannel activity, reduced cell tolerance to oxidative stress, changed the protein distribution pattern and inhibited the cell growth.

**Conclusions:**

We have identified a novel missense mutation in *GJA8* (c.205G > A, p.Ala69Thr) in a four-generation Chinese family and our results will further broaden the gene mutation spectrum of *GJA8*.

**Supplementary Information:**

The online version contains supplementary material available at 10.1186/s12886-020-01725-1.

## Background

Congenital cataract is defined as a type of lens opacification that occurs at birth or at an early age, which could damage the vision development. It is estimated that the global prevalence of congenital cataract is 4.24 per 10,000 live births [1]. In industrialized countries, the prevalence of congenital cataract is 1 to 6 per 10,000, while in China, the prevalence of congenital cataract is about 5 cases per 10,000 [2]. The incidence of congenital cataract is related to many factors, and hereditary factor is the most important one and responsible for the majority of cases [3–5]. The most common genetic pattern of congenital cataract is autosomal dominant, and there are also reports of autosomal recessive and X-linked forms. The known mutant genes responsible for congenital cataracts include lens membrane proteins (*GJA3*, *GJA8*, *MIP*), crystallins (*CRYAA*, *CRYAB*, *CRYBA1/A3*, *CRYBA4*, *CRYBB1*, *CRYBB2*, *CRYGC*, *CRYGD* and *CRYGS*), cytoskeletal proteins (*BFSP1*, *BFSP2*), growth and transcription factors (*HSF4*) and others [6–11]. Among these mutant genes, the crystalline genes and connexin genes are the most widespread, which are involved in most of reported congenital cataract cases [12].

Connexins (Cxs), clusters of membrane-spanning proteins, consist of two extracellular loops and a cytoplasmic loop that connect four transmembrane domains with the NH2-terminus and the COOH-terminus in the cytoplasm [13]. Connexins compose gap junctions, which are membrane specializations that permeable to ions and small metabolites and could behave as a functional syncytium (≤1 kDa) [14, 15]. Gap junctions mediate direct cell-cell interaction and have a great effect on the normal function and survival of cells to maintain the transparency and homeostasis of lens [16, 17]. Six connexin subunits in the plasma membrane of two adjacent cells compose hemichannel and two hemichannels form one channel [18]. Different channels, due to the properties of the specific Cxs, play different roles including direct exchange, penetration, chemical gating and voltage-dependence gating [19–22]. These functions play a significant role in maintaining the normal metabolism of the lens.

In this work, we investigated a four-generation Chinese family affected with autosomal dominant congenital cataract. By target region sequencing, we identified a novel missense mutation in gap junction protein alpha-8 (*GJA8*), which substituted threonine for alanine (p.Ala69Thr), and explored the functional impact of the mutation.

## Methods

### Patients

This study was approved by the medical ethics committee of Tianjin Medical University Eye Hospital and was in compliance with regulations of the Declaration of Helsinki of the World Medical Association. A four-generation Chinese family was recruited and written informed consent was obtained after explanation of the nature and possible consequences of the study. The enrolled subjects were examined by the professional ophthalmologist. A 5 ml venous blood sample of each patient was drawn into an ethylenediamine tetraacetic acid (EDTA) sample tube.

### DNA sequencing and bioinformatics analysis

Genomic DNA was extracted according to the manufacturer’s standard procedure (MagPure Buffy Coat DNA Midi KF Kit, Magen, China) and the genomic DNA of the proband was sequenced on MGISEQ-2000 (PE100). The targeted sequences were captured using the BGI Exome V4 chip, which contained 527 genes related to eye diseases according to OMIM. All potential pathogenic variants were validated using conventional Sanger sequencing methods. Segregation analysis was performed in all available family members. The structures of homomeric wild-type and the mutant *GJA8* were modeled by Swiss-Model Server (https://swissmodel.expasy.org) and shown using a PyMOL Molecular Graphic system, using the solved structure of connexin-50 (Cx50)-protein coded by *GJA8* gene as template (Protein Data Bank No.6MHY_A). Multiple sequence alignment of Cx50 sequences from different species was performed by CLUSTALW (https://www.genome.jp/tools-bin/clustalw). In addition, the possible functional effect of the amino acid change was predicted by PolyPhen-2 (http://genetics.bwh.harvard.edu/pph2/) and Provean (http://provean.jcvi.org/index.php).

### Cell lines and cell culture

Chicken embryonic fibroblast DF-1 cells obtained from the American Type Culture Collection (CRL-12203, ATCC, Maryland, USA) were used to perform functional analysis. The cells were cultured at 37°C in a humidified atmosphere containing 5% CO_2_ in high glucose Dulbecco’s Modified Eagle Medium (4.5 g D-Glucose/L, Basal Media, Shanghai, China) supplemented with 100 U/ml penicillin, 10% fetal bovine serum and 100 μg/ml streptomycin (Gibco, Thermo Fisher Scientific, Inc., Waltham, MA, US).

### Lentiviral plasmid and gene transfection

Lentiviral plasmids containing wild-type *GJA8* and mutant *GJA8*Ala69Thr gene and vector plasmids were synthesized by Biogot Technology, Co. Ltd. (Nanjing, China). All constructs were verified by nucleic acid sequencing. According to the transfection protocols, the plasmids were transfected into the DF-1 cells with 20–30% confluence (5 × 10^5^ cells) in a 60-mm dish. The effect of gene transduction was verified by western blotting. The transfection efficiency of lentiviral plasmids in these cells was verified by western blotting.

### Western blotting

Total protein was extracted using RIPA buffer (Solarbio, Beijing, China) with 20 mM NEM and 1 mM PMSF from cells. The protein concentration was analyzed by BCA assay (Thermo Scientific Inc.) and western blotting was performed by 4–15% SDS-PAGE (Bio-Rad Laboratories, Hercules, CA, USA). The proteins were transferred onto polyvinylidene difluoride membranes (Bio-Rad Laboratories) after electrophoresis, and then blocked with 5% skim milk powder and 0.1% TBS-Tween for 1 h at room temperature. After incubation with anti-*GJA8* (ab222885; Abcam; 1:2000) or anti-β-actin (ab8227; Abcam; 1:1000) primary antibodies at 4°C overnight, the membranes were washed 5 times in 0.1% TBS-Tween and incubated for 1 h with a chicken anti-rabbit IgG horseradish peroxidase-conjugated secondary antibody (dilution, 1:2000; cat. no. sc-516,087; Santa Cruz Biotechnology, Inc., Dallas, TX, USA) at room temperature. Super Signal protein detection kit (Pierce; Thermo Fisher Scientific, Inc.) was used to detect labeled proteins and ImageJ software (National Institutes of Health, Bethesda, MD, USA) was used to evaluate protein levels changes.

### Dye uptake assay

Hemichannel activity was traced by 4 μM ethidium bromide (EtBr) [23]. 1 × 10^5^ DF-1 cells with *GJA8* or mutant *GJA8*Ala69Thr or vector transgene were grown for 12 days at a low-cell density. Then cells were treated with 0.3 mL 0.5 mM H_2_O_2_ for 2 h and were rinsed three times with Hanks’ balanced salt solution (HBSS). After that, cells were fixed with 2% PFA for 30 min. Four fluorescence fields microphotographs were taken with a 20 × dryobjective in Olympus aninverted microscope with a Rhodamine filter. ImageJ software was used to measure the average pixel density of 30 random DF-1 cells.

### Immunofluorescence

5 × 10^5^ DF-1 cells containing *GJA8* or mutant *GJA8*Ala69Thr plasmid or vector transgene were cultured for 48 h on glass coverslips. Then the cells were fixed with 4% paraformaldehyde (Sigma-Aldrich; Merck KGaA), permeabilized for 5 min in 0.5% Triton X-100, blocked for 1 h with 3% bovine serum albumin (Sigma-Aldrich) at room temperature. Subsequently, cells were incubated overnight at 4°C with anti-*GJA8* (1:100, ab222885, Abcam), and further incubated for 1 h at room temperature with Alexa Fluor® 594-conjugated goat anti-rabbit IgG (1:500, ab150080, Abcam). A confocal microscope (Carl Zeiss AG, Oberkochen, Germany) was used to observe protein expression and subcellular localization.

### Determination of dead cells

DF-1 cells were exposed to 1 mL 0.5 mM H_2_O_2_ for 10 h when they grew to 90% confluence, and were trypsinized and collected subsequently. Using the Dead Cell Apoptosis Kit (BioLegend, San Diego, CA), Annexin V-FITC staining was examined in cell suspension according to manufacturer’s instructions. At least three random fields were examined under a fluorescence microscope for each condition treated.

### Colony formation assays

2 × 10^3^ DF-1 cells with *GJA8* or mutant *GJA8*Ala69Thr or vector transgene were seeded in 6-well plates. The cells were fixed with 4% paraformaldehyde for 30 mins after 14 days of culture. The colonies were stained with 5% crystal violet for 30 mins after washing with PBS again. Colonies exceeding 50 cells were counted using ImageJ. Twenty clones in each group were randomly selected, their diameters were measured, and the averages were compared by appropriate statistical methods. Each group consisted of three duplicates, and the experiment was performed three times.

### Statistical analysis

The data were analyzed using GraphPad Prism 6 (GraphPad Software, Inc.). Comparisons between two groups were analyzed using Student’s t-test. Differences among multiple groups was analyzed by one-way ANOVA followed by Tukey’s post hoc test. *P* < 0.05 was considered to indicate statistically significant differences. All experiments were repeated at least three times.

## Results

### Clinical evaluation

**Fig. 1:**
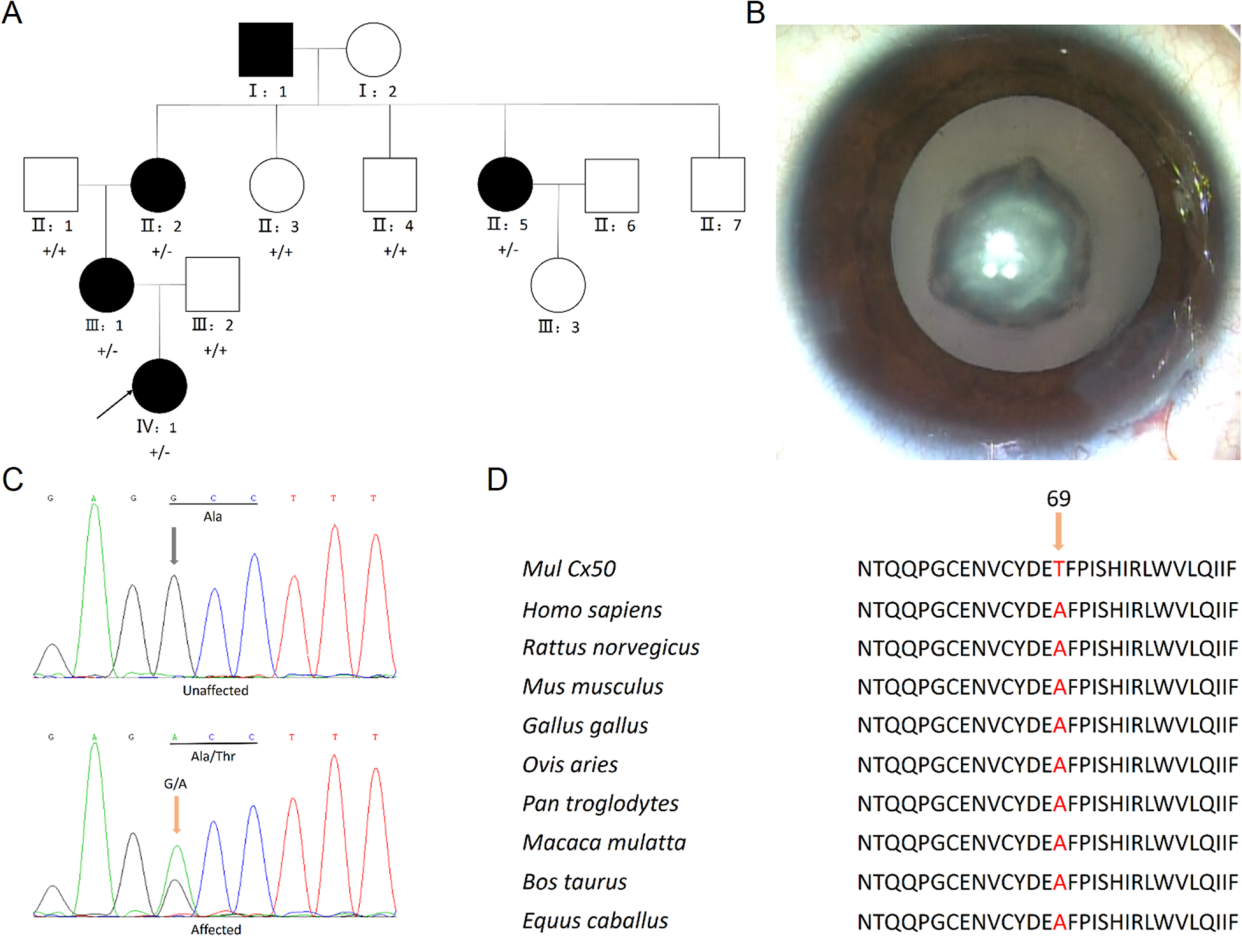
Clinical evaluation and mutation identification of a four-generation Chinese family with congenital cataract. **a** Pedigree map of the family. The arrow indicates the proband. Squares and circles symbolize males and females, respectively. Black and white denotes affected and unaffected individuals, respectively. + represents wild-type *GJA8* allele; − represents allele with mutation. **b** Snapshot during surgery of the proband showed nuclear cataract. **c** Sanger sequencing of *GJA8* detected a c.205G > A transversion in affected patients which caused the replacement of a wild-type alanine with threonine at codon 69 (p.Ala69Thr). **d** Multiple-sequence alignments of *GJA8* in various species showed codon 69 was located within a highly conserved region

Four patients (II:2, II:5, III:1, IV: 1) and four normal individuals (II:1, II:3, II:4, III:2) from this family were enrolled in this study (Fig. 1a). The proband was a five-year-old girl (IV: 1) and was diagnosed with bilateral nuclear congenital cataract (Fig. 1b). On presentation, the visual acuity of both eyes was 20/200. The axial length was 20.06 mm and 20.02 mm, the anterior chamber depth was 2.43 mm and 2.62 mm, the lens thickness was 4.27 mm and 4.22 mm, the flat K was 39.76D and 40.18 D, the steep K was 41.16 D and 42.47 D for the right eye and left eye, respectively. The other 3 affected individuals were diagnosed congenital nuclear cataract and accepted cataract surgery several years ago. Other ocular or systemic abnormalities have not been found in this family. There was no consanguineous marriage in this family. The detailed clinical information of the enrolled individuals is displayed in Table 1.

**Table 1:** Clinical information of enrolled individuals

Individuals	Age at recruitment (years)	Age at diagnosis (years)	Age at cataract surgery (years)	IOP (mmHg) at recruitment (R, L)	BCVA at recruitment (R, L)
II:2	55	4	10	21.2, 20.4	20/40, 20/50
II:5	47	2	6	15.6, 14.3	20/30, 20/30
III:1	29	1.5	6	14.8, 16.2	20/25, 20/20
IV: 1	5	2	5	12.3, 11.4	20/200, 20/200
II:1	55	NA	NA	14.6, 17.8	20/25, 20/20
II:3	52	NA	NA	17.1, 14.9	20/20, 20/25
II:4	49	NA	NA	19.1, 15.8	20/20, 20/20
III:2	31	NA	NA	11.2, 16,3	20/20, 20/20

*IOP* Intraocular pressure, *BCVA* Best corrected visual acuity, *NA* Not applicable, *R* Right eye, *L* Left eye

### Mutation identification in *GJA8*

Targeted exome sequencing containing 527 eye diseases-related genes of the proband (IV: 1) revealed a transversion in exon 2 (c.205G > A) of *GJA8* and a frameshift deletion in exon 2 (c.37delC) of *TMEM138* without any change in the remainder of the coding sequence. The mutations of *TMEM138* are reported to be responsible for Joubert syndrome, which is inconsistent with the phenotype of our family. By further Sanger sequencing, the mutation (c.37delC) of *TMEM138* was not found either in the unaffected family members or in other affected family members. Therefore, we do not think the mutation of *TMEM138* detected in the proband is responsible for this congenital cataract family. The *GJA8* mutation causes the replacement of a wild-type alanine with threonine at codon 69 (p.Ala69Thr; Fig. 1c). The mutation can be detected in all affected patients enrolled in this study (II:2, II:5, III:1, IV: 1) and was not found in the unaffected family members (II:1, II:3, II:4, III:2) by further Sanger sequencing.

### Bioinformatics analysis

**Fig. 2:**
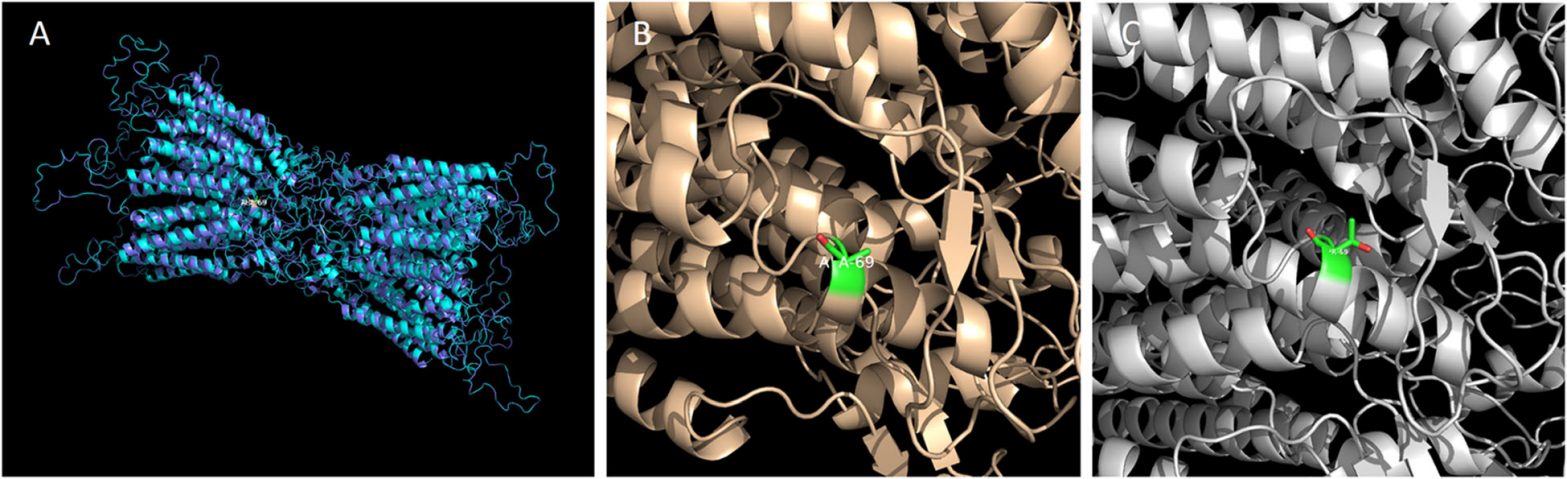
Structure homology modeling of *GJA8*. **a** Side view of overlapped wild-type and p.Ala69Thr mutant *GJA8* in cartoon form, showing little change on the overall structure of the protein. **b** Highlighted codon 69 amino acid side chain of wild-type *GJA8*. **c** Highlighted codon 69 amino acid side chain of p.Ala69Thr mutant *GJA8*

Multiple-sequence alignments of *GJA8* in various species showed codon 69 was located within a highly conserved region (Fig. 1d). The online bioinformatics software PolyPhen-2 indicated the mutation was “probably damaging” with a score of 1.0. Provean gave a score of − 2.704, which predicted a “deleterious” effect of the mutation. The homology modeling showed no evident change on the overall structure of the protein (Fig. 2a), the amino acid conformation was not altered either except for some side chains difference of Thr and Ala (Fig. 2b, c).

### Functional analysis

**Fig. 3:**
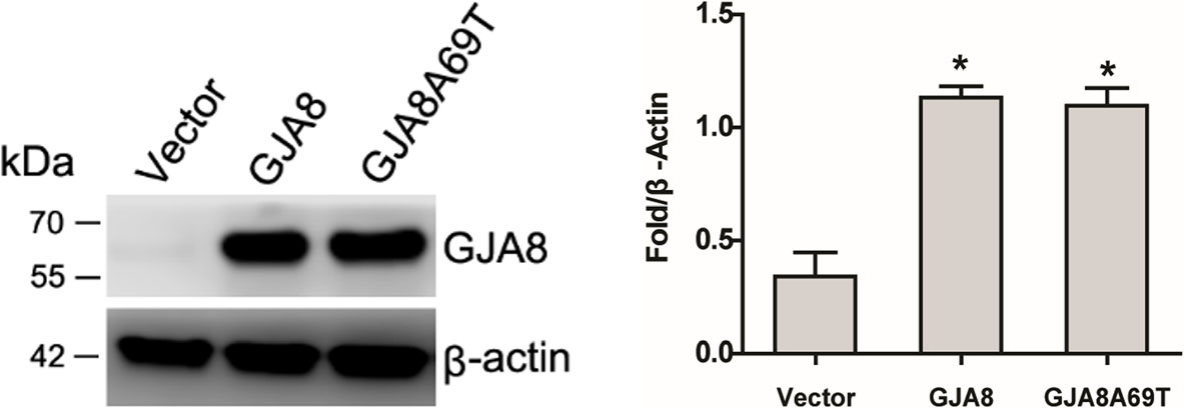
Western blot verified successful transfection of DF-1 cells with wild-type *GJA8* and mutant *GJA8*Ala69Thr gene

We got the successful gene transfection confirmed by western blot (Fig. 3, supplementary figure 1).

**Fig. 4:**
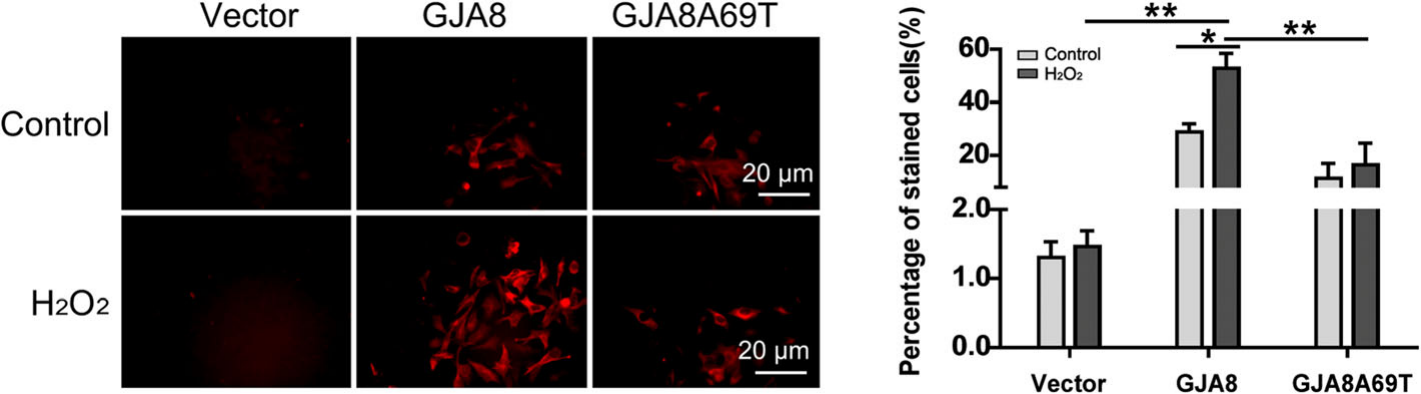
Dye uptake assay. After H_2_O_2_ treatment, EtBr uptake was increased in *GJA8*- expressing cells but not in cells expressing mutant *GJA8*A69T (Student’s t-test and ANOVA, * *p* < 0.05, ** *p* < 0.01)

The EtBr dye uptake assay was used to determine the hemichannel activity change. From Fig. 4, we found no increase in dye absorption after treatment with H_2_O_2_ in *GJA8*A69T cells while the dye uptake increased after H_2_O_2_ treatment in *GJA8* cells, indicating the hemichannel activity was inhibited by the mutation.

**Fig. 5:**
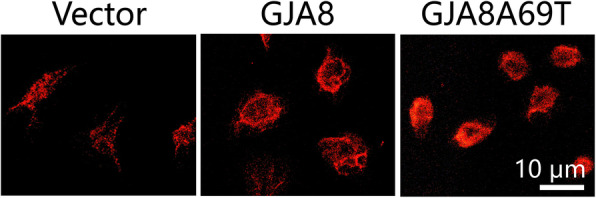
Immunofluorescent imaging showed more protein accumulation in the cytoplasm in mutant *GJA8*Ala69Thr cells

Confocal immunofluorescent analysis showed that the Cx50 proteins expressed by cells transfected with wild-type *GJA8* were mainly present on the cell membrane and the p.Ala69Thr mutation resulted in presence of more Cx50 protein in cytoplasm (Fig. 5), indicating that Cx50 p.Ala69Thr protein caused a mislocalization and translocation into cytoplasm.

**Fig. 6:**
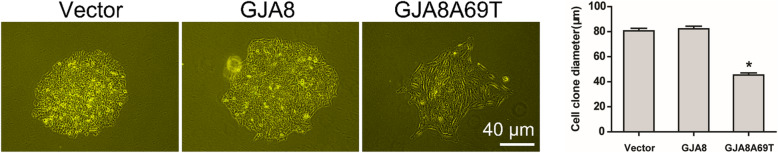
Colony formation assays showed the colony size of mutated *GJA8*Ala69Thr group was much smaller than control groups (ANOVA, * *p* < 0.05)

The cell colony assays showed a smaller colony size of mutant p.Ala69Thr cells, compared with wild-type control (Fig. 6), indicating the mutation inhibited cell growth in vitro.

**Fig. 7:**
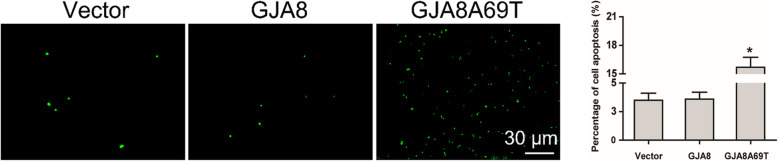
Annexin V-FITC staining assay showed more cell death in DF-1 cells expressing GJA8Ala69Thr than control groups (ANOVA, * *p* < 0.05)

The Annexin V-FITC staining assay showed more cell death in mutant cells than wild type cells after H_2_O_2_ (Fig. 7), indicating the mutant cells had a reduced tolerance to oxidative stress induced by H_2_O_2_ treatment.

## Discussion

In this study, by targeted sequencing (527 genes related to eye diseases according to OMIM were included) and Sanger sequencing, a novel heterozygous mutation (c.205G > A; p.Ala69Thr) within *GJA8* was identified. Function analysis indicated that the mutation inhibited GJA8 hemichannel activity, reduced cell tolerance to oxidative stress, changed the protein distribution pattern and inhibited the cell growth.

Cx50, encoded by *GJA8*, has its unique characteristics. The hemichannel formed by Cx50 is monovalent cation sensitive and more sensitive to extracellular acidification [12]. Studies also indicated that the Cx50 gap junction gates have positive relative polarity and Cx50 hemichannel currents could be reversibly blocked by the histidine modifier, diethyl pyrocarbonate [24]. Recent studies have shown that Cx50 hemichannels possibly protect lens fiber cells from oxidative stress damage by transporting extracellular reductants [23]. However, the specific function and regulatory mechanism of Cx50 hemichannel in the lens is still unclear.

In our current study, we report that the p.Ala69Thr mutation, located in the first extracellular loop, is related to congenital cataract, examined by bioinformatics and functional analysis. Nearly 70 different mutations in *GJA8* gene have been discovered so far (https://cat-map.wustl.edu/), and about half of the mutations are located in extracellular loops, indicating the two extracellular loops are mutational hot spots [7, 25]. Several mutations have been identified near position 69 of Cx50 that are associated with congenital cataract, such as p.V64G, p.D67G, p.S73F, p.R76C, and p.V79L [26], suggesting that the A69 residue and surrounding region may play an important role in maintaining normal Cx50 function. However, the genotype-phenotype correlation for inherited cataract is still unclear, which needs further studies to confirm.

Although the homology modeling showed no evident change on the overall structure of the protein except for some side chains difference of Thr and Ala, the p.Ala69Thr mutation was predicted to be deleterious by both PolyPhen-2 and Provean with consistent results. Codon 69, where the mutation occurred, is also found to be located within a highly conserved region by multiple sequence alignments. Taken together, these data suggest that the p.Ala69Thr substitution is a causative disease mutation rather than a simple polymorphism.

Connexin hemichannels are normally inactive and are activated in response to certain stimuli and cell stress. In order to elucidate the effect of oxidative stress on connexin hemichannel activity, we infected chicken embryonic fibroblast cells with lentiviral plasmids containing wild-type *GJA8* and mutant *GJA8*Ala69Thr, and treated the cells with H_2_O_2_. Our results showed EtBr dye uptake was increased in *GJA8* cells after H_2_O_2_ treatment, indicating the hemichannels were successfully activated. However, for *GJA8*Ala69Thr cells, dye uptake was not increased after H_2_O_2_ treatment, suggesting the mutation inhibited Cx50 hemichannel activity, which will further attenuate extracellular reductants transportation [23]. The inhibition of hemichannels can also increase the susceptibility of the cells from oxidative stress. Our Annexin V-FITC staining assay showed more cell death in mutant cells than wild type cells after H_2_O_2_ treatment, indicating the mutation reduced cell tolerance to H_2_O_2_-induced oxidative stress, which is proved to be the major factor contributing to the development of cataract, both in vitro *and* in vivo [27–29].

The distribution of Cx50 protein was also affected by p.Ala69Thr mutation. Our immunofluorescent results showed that in *GJA8*Ala69Thr cells, more mutant protein was accumulated in the cytoplasm while Cx50 protein was mainly distributed in plasma membrane in *GJA8* cells, indicating the mutation led to protein accumulation and caused changes in Cx50 protein localization pattern. The increased accumulation and the different distribution pattern of the mutant protein may alter the function of gap channels and unbalance the metabolism of lens fiber, eventually lead to cataract formation [25]. We found a negative effect of p.Ala69Thr mutation on cell growth, which is different from the study of Ge et al [7]. In their study, they found a positive effect of p.P88T mutation on cell growth. Although the underlying mechanism remains unclear, the distinct results highlight the high genetic heterogeneity of congenital cataract. Different mutations at the same residue render different phenotypes and different mutations lead to cataract formation by different mechanisms.

## Conclusions

In conclusion, we have identified a novel missense mutation in GJA8, p.Ala69Thr, in a four-generation Chinese family with multiple individuals having autosomal dominant congenital cataract. The mutation inhibited the Cx50 hemichannel activity, reduced cell tolerance to oxidative stress, changed the protein distribution pattern and inhibited the cell growth in vitro, which may account for the underlying molecular mechanisms in this congenital cataract family. Our results can broaden the gene mutation spectrum of congenital cataract, enrich the pathogenic mechanism of congenital cataract, and may provide therapeutic target for possible gene therapy in the future.

## Supplementary Information

Additional file 1 : **Supplementary figure 1.** The expression of GJA8 (left panel) and actin (right panel) was detected by western blot.

## Data Availability

Data used/generated by this study are available from the corresponding author upon reasonable request.

## Abbreviations

Cxs: Connexins
GJA8: Gap junction protein alpha-8
Cx50: Connexin-50
EtBr: Ethidium bromide
