# Supplementary figures and images for: Identification and functional analysis of a novel missense mutation in GJA8, p.Ala69Thr
Source: BMC Ophthalmol. 2020 Nov 20;20:461. doi: 10.1186/s12886-020-01725-1 (PMC7678044; doi:10.1186/s12886-020-01725-1)

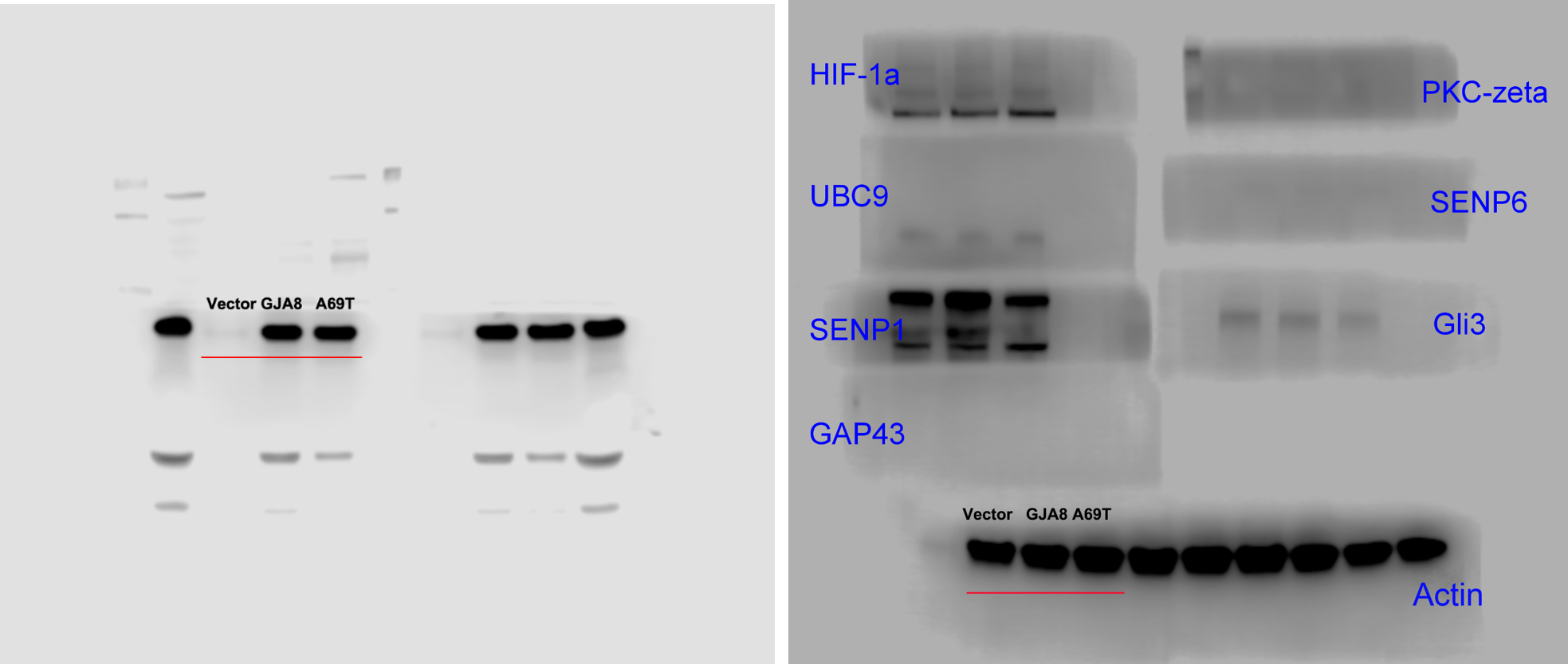

Supplement: Supplementary file 1 — : Supplementary figure 1. The expression of GJA8 (left panel) and actin (right panel) was detected by western blot. [file 12886_2020_1725_MOESM1_ESM.tif]
